# Prognostic biomarker identification and tumor classification in breast cancer patients by methylation and transcriptome analysis

**DOI:** 10.1002/2211-5463.13211

**Published:** 2021-06-25

**Authors:** Xiongdong Zhong, Guoying Zhong

**Affiliations:** ^1^ Department of Cardiothoracic Surgery Zhuhai People's Hospital (Zhuhai hospital affiliated with Jinan University) China

**Keywords:** breast cancer, DNA methylation, immunohistochemical staining, prognosis, tumor classification

## Abstract

Breast cancer is one of the most common and heterogeneous malignancies. Although the prognosis of breast cancer has improved with the development of early screening, the mechanisms underlying tumorigenesis and progression remain incompletely understood. DNA methylation has been implicated in tumorigenesis and tumor development and, in the present study. we screened methylation‐driven genes and explored their prognostic values in breast cancer. RNA‐sequencing (RNA‐Seq) transcriptome data and DNA methylation data of the TCGA‐BRCA dataset were obtained from The Cancer Genome Atlas. Differentially expressed genes and differentially methylated genes were identified separately. The intersected 783 samples with both RNA‐Seq data and DNA methylation data were selected for further analysis. Fifty‐six methylation‐driven genes were identified using the MethylMix r package and 10 prognosis methylation‐driven genes (*CDO1*, *CELF2*, *ITPAIPL1*, *KCNH8*, *PTK6*, *RAB25*, *RIC3*, *USP44*, *ZSCAN1* and *ZSCAN23*) were further screened by combined methylation and gene expression analysis. Based on the methylation data of the screened 10 methylation‐driven genes, six subgroups were identified with the ConsensusClusterPlus r package. The protein levels of the 10 prognostic methylation‐driven genes were detected by immunohistochemical experiments. Moreover, based on the RNA‐Seq data, a signature calculating the risk score of each patient was developed with stepwise regression. The risk score and other clinical features (age and stage) were confirmed to be independent prognostic factors by univariate and multivariate Cox regression analyses. Finally, a prognostic nomogram incorporating all the significant factors was integrated to predict the 3‐, 5‐ and 7‐year overall survival. Taken together, the methylation‐driven genes identified here may be potential biomarkers of breast cancer.

## Graphical abstract

DNA methylation has been implicated in tumorigenesis and tumor development. In the present study, we screened methylation‐driven genes and explored their prognostic values in breast cancer by combined methylation and gene expression analysis. The methylation‐driven genes identified may be potential biomarkers of breast cancer.

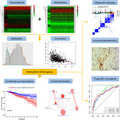

**Abbreviations**
BC: breast cancer
FDR: false discovery ratio
GO: Gene Ontology
KEGG: Kyoto Encyclopedia of Genes and Genomes
RNA‐Seq: RNA sequencing
ROC: receiver operating characteristic
TNBC: triple negative breast cancer

Breast cancer (BC) is one of the most common and heterogeneous malignancy and has become the main cause of cancer‐associated death among females in the world [1, 2]. Clinically, the subtypes of BC are commonly considered to include ER^+^, PR^+^, HER2^+^ and triple negative breast cancer (TNBC) [3]. Although the therapeutic methods and prognosis of BC have been significantly improved with the development of early screening, molecular genetics and targeted therapies, the mechanisms of the tumorigenesis and progression still remain relatively unknown.

Genomic instability has a very important role in the tumorigenesis and development of carcinogenesis [4]. DNA methylation is one type of epigenetic modification associated with gene expression and genomic stability [5]. The alterations in DNA methylation comprise early events in carcinogenesis, which is of great clinical interest for potential biomarkers with respect to diagnosis, prognosis, therapeutic classification and follow‐up after treatment [6]. For example, methylation of the promoter for *O* ^6^‐methylguanine‐DNA methyltransferase has been well studied and included in the NCCN clinical practice guidelines to determine the therapeutic method in glioblastoma [7]. The clinical value of plasma septin9 for the detection of asymptomatic colorectal cancer was revealed and promoted an early diagnosis [8]. The methylation level of serum biomarker EFC#93 was identified as a new way of diagnosing and managing BC [9]. To date, our understanding of methylation‐driven genes in BC is still lacking.

MethylMix is a bioinformatic tool applied to screen hyper and hypo methylated genes [10]. According to a beta mixture model, it screens methylation status and compares this with the normal DNA methylation state [11]. The negative correction between DNA methylation and RNA expression is also considered and calculated to improve the accuracy. ConsensusClusterPlus also comprises a bioinformatic tool widely used in studies of tumor classification [12].

In the present study, the MethylMix r package was used to identify the methylation‐driven genes. Then, combined methylation and gene expression analyses were performed to screen prognosis biomarkers. Consensus clustering was applied with the ConsensusClusterPlus r package to identify subgroups. Finally, the protein levels of the prognosis methylation‐driven genes were detected by immunohistochemical experiments. In total, 56 methylation‐driven genes were identified, of which 10 survival‐related genes were further explored in the molecular tumor classification. Moreover, based on the RNA‐sequencing (RNA‐Seq) data, a signature calculating the risk score of each patient was developed with stepwise regression. The risk score and other clinical features (Age and Stage) were confirmed to be independent prognostic factors by univariate and multivariate Cox regression analyses. Finally, a prognostic nomogram incorporating all the significant factors was integrated to predict the 3‐, 5‐ and 7‐year overall survival. Taken together, the screened methylation‐driven genes could be potential biomarkers of BC.

## Materials and methods

### Data resources and analysis

RNA‐Seq data (fragments per kilobase of transcript per million mapped reads values) of the TCGA‐BRAC dataset and corresponding clinical information were obtained from The Cancer Genome Atlas (cancergenome.nih.gov), involving 113 normal and 1109 tumor samples. DNA methylation data (beta value) ranging from 0 to 1 (unmethylated to totally methylated) of the TCGA‐BRCA dataset were also downloaded from The Cancer Genome Atlas, including 96 normal and 796 tumor samples. First, differentially expressed genes were screened on the basis of a Wilcox test in r, version 3.6.1 (R Foundation for Statistical Computing, Vienna, Austria) with a false discovery ratio (FDR) < 0.05 and absolute log2 fold change > 1. DNA methylation data were merged using Perl, version 5.32.0 (https://www.perl.org/get.html) and differentially methylated genes were identified using a Wilcox test with FDR < 0.05 and absolute log_2_ fold change > 0.5. Methylation‐driven genes are those genes for which the DNA methylation levels are negatively correlated with the mRNA expression level after linear regression analysis. Then, the intersected 783 samples including both RNA expression data and DNA methylation data were selected. The MethylMix r package was used to identify the methylation‐driven genes with Pearson correlation between the DNA methylation level and RNA expression < −0.3 and *P* < 0.05. The methylation mixture models were plotted via the MethylMix_PlotModel function within the MethylMix r package. Finally, a total of 56 methylation‐driven genes were obtained. The heatmaps of RNA expression and the methylation level of 56 methylation‐driven genes were plotted using the r pheatmap package in r, version 3.6.1.

### Functional enrichment analysis

To reveal the function of the methylation‐driven genes, Gene Ontology (GO) enrichment analysis was performed with the clusterProfiler r package and Kyoto Encyclopedia of Genes and Genomes (KEGG) pathway enrichment analysis was applied via ConsensusPathDB (http://cpdb.molgen.mpg.de). *P* < 0.05 was used to distinguish significantly enriched terms.

### Combined gene expression and methylation survival analysis

Gene expression and methylation survival analyses were combined to identify potential biomarkers to predict the prognosis. In total, 754 samples with an overall survival of between 30 and 3650 days were selected into the survival analysis at beginning. According to the median levels of the methylation data and RNA expression data of each gene, patients were divided into hyper methylation, hypo methylation, high expression and low expression groups. For each gene, only the patients with hyper methylation and low expression or hypo methylation and high expression were included into the combined survival analysis. Kaplan–Meier curve analysis was further performed to find prognosis genes with *P* < 0.05.

### Molecular subtypes related to prognosis

Consensus clustering was conducted with the ConsensusClusterPlus r package in r, version 3.6.1 to screen BRCA subgroups according to the methylation data of the prognosis methylation‐driven genes. The algorithm started with subsampling from the methylation data and every subsample was separated into *k* groups by *k*‐means. This was repeated for 100 times to reach consensus values and get the stability of the screened clusters. After ConsensusClusterPlus was applied, the cluster consensus and item‐consensus results were obtained.

### Analysis of subgroups in survival and clinical features

Survival analysis of subgroups were performed with the survival r package. The differences among the clusters were indicated with Kaplan–Meier plots. Boxplots of methylation levels were performed using the reshape2 r package. Associations between clinical features (age, TNM, stage) were plotted and different methylation level among clusters were calculated by a Wilcox test with FDR < 0.05 and delta beta > 0.1. The difference was plotted using the pheatmap r package.

### Immunohistochemistry (IHC)

For the purpose of revealing the different protein expression levels of the prognosis methylation‐driven genes, immunohistochemical experiments were performed. In total, 42 tumor and adjacent normal tissues were collected from our hospital. The study was approved by the Research Ethics Committee of the Zhuhai People's Hospital and informed written consent was obtained from each patient. Methodologies in the present study conformed to the standards set by the Declaration of Helsinki. All the tissues were fixed with 4% neutral formaldehyde for 24 h. Sections (4 μm) were generated after dehydration and paraffin embedding. Incubation with primary antibodies including rabbit CDO1 antibody (dilution 1:200; Abcam, Cambridge, MA, USA), rabbit CELF2 (ETR3) antibody (dilution 1:100; Abcam), rabbit ITPRIPL1 antibody (dilution 1:200; Biolab, Beijing, China), rabbit KCNH8 antibody (dilution 1:200; Biolab), rabbit PTK6 antibody (dilution 1:200; Abcam), rabbit RAB25 antibody (dilution 1:200; Biolab), rabbit RIC3 antibody (dilution 1:100; Abcam), rabbit USP44 antibody (dilution 1:200; Abcam), rabbit ZSCAN1 antibody (dilution 1:200; Abcam) and rabbit ZSCAN23 antibody (dilution 1:200; Abcam) was conducted overnight at 4 °C. Phosphate‐buffered saline without primary antibody was used as the negative control. DAB chromogenic reagent was applied to develop the stain and hematoxylin was used to stain the nucleus. The sections were finally dehydrated and mounted with a neutral resin onto slides. Digital photomicrographs of sections were taken from representative areas at a fixed magnification of 200×. Positive staining in images was quantified as the integral optical density/area, which was expressed as the mean density using image‐pro plus, version 6.0 (Media Cybernetics, Inc., Bethesda, MD, USA). Then, the mean density values were analyzed with prism, version 8 (GraphPad Software Inc., San Diego, CA, USA). A paired *t*‐test was conducted to compare the differential expressions between tumor and normal tissues.

### Integration and evaluation of the prognostic nomogram

To further explore the prognostic value of the 10 identified survival‐related and methylation‐driven genes, multivariate Cox regression was performed to establish a nomogram. First, a signature estimating the risk score of each patient was constructed with stepwise regression based on the RNA‐Seq data. Then, the independence of the signature and the other clinical features (Age and Stage) was confirmed by univariate and multivariate Cox regression analyses with the survival r package. The hazard ratio and *P*‐values were plotted. Finally, a prognostic nomogram incorporating all the significant factors (*P* < 0.05) was integrated to predict the 3‐, 5‐ and 7‐year overall survival with the rms r package. The receiver operating characteristic (ROC) 3‐, 5‐ and 7‐year curves were plotted with the survivalROC r package and the calibration were also carried out using the rms r package to show the prognostic predictive accuracy of the nomogram.

## Results

### Screening of methylation‐driven genes in BRCA

**Fig. 1:**
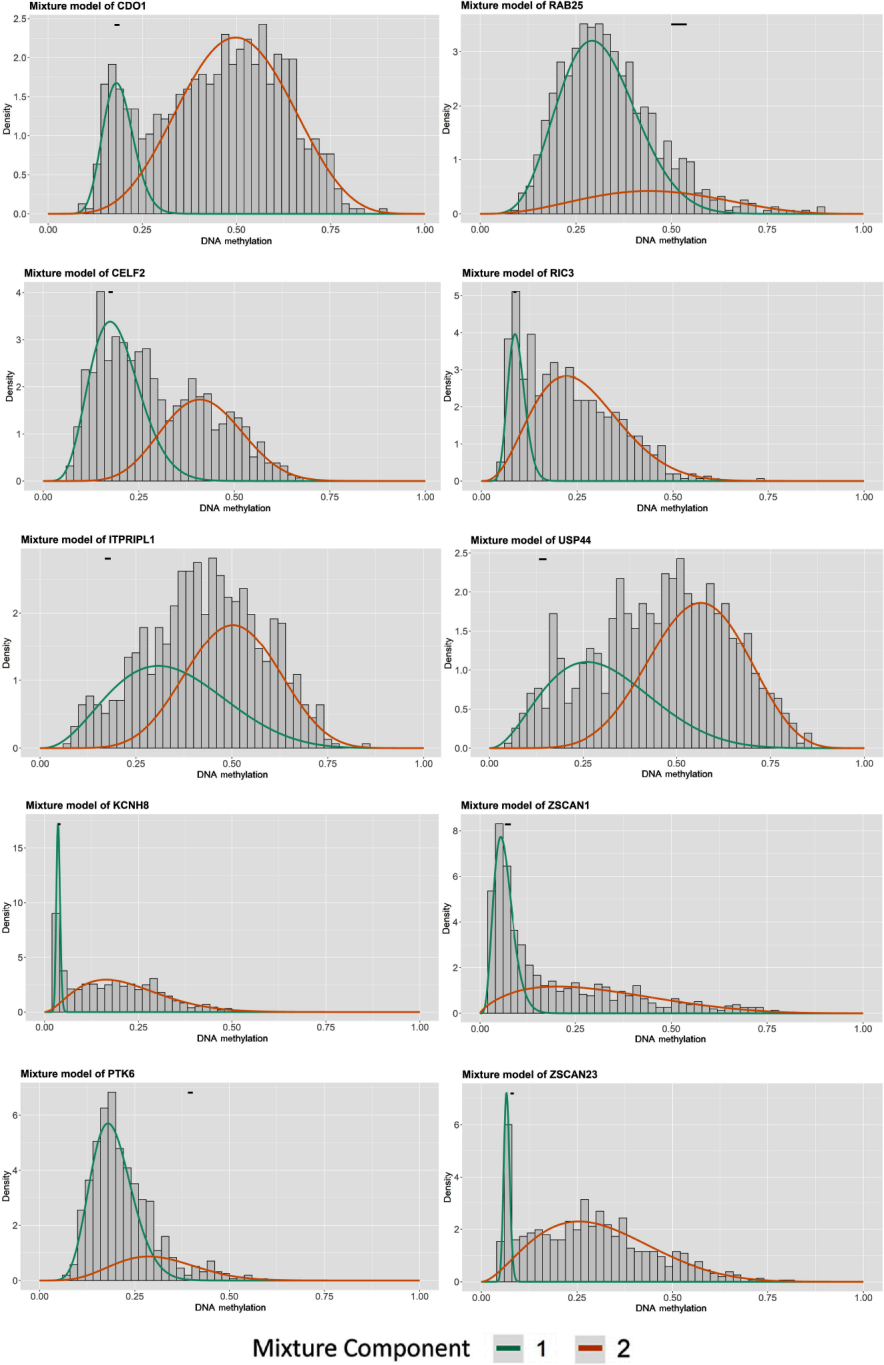
The methylation mixture models plotted by MethylMix in BRCA. The red and green curves indicate the methylation level of the cancer and the black line is the distribution in normal samples.

**Fig. 2:**
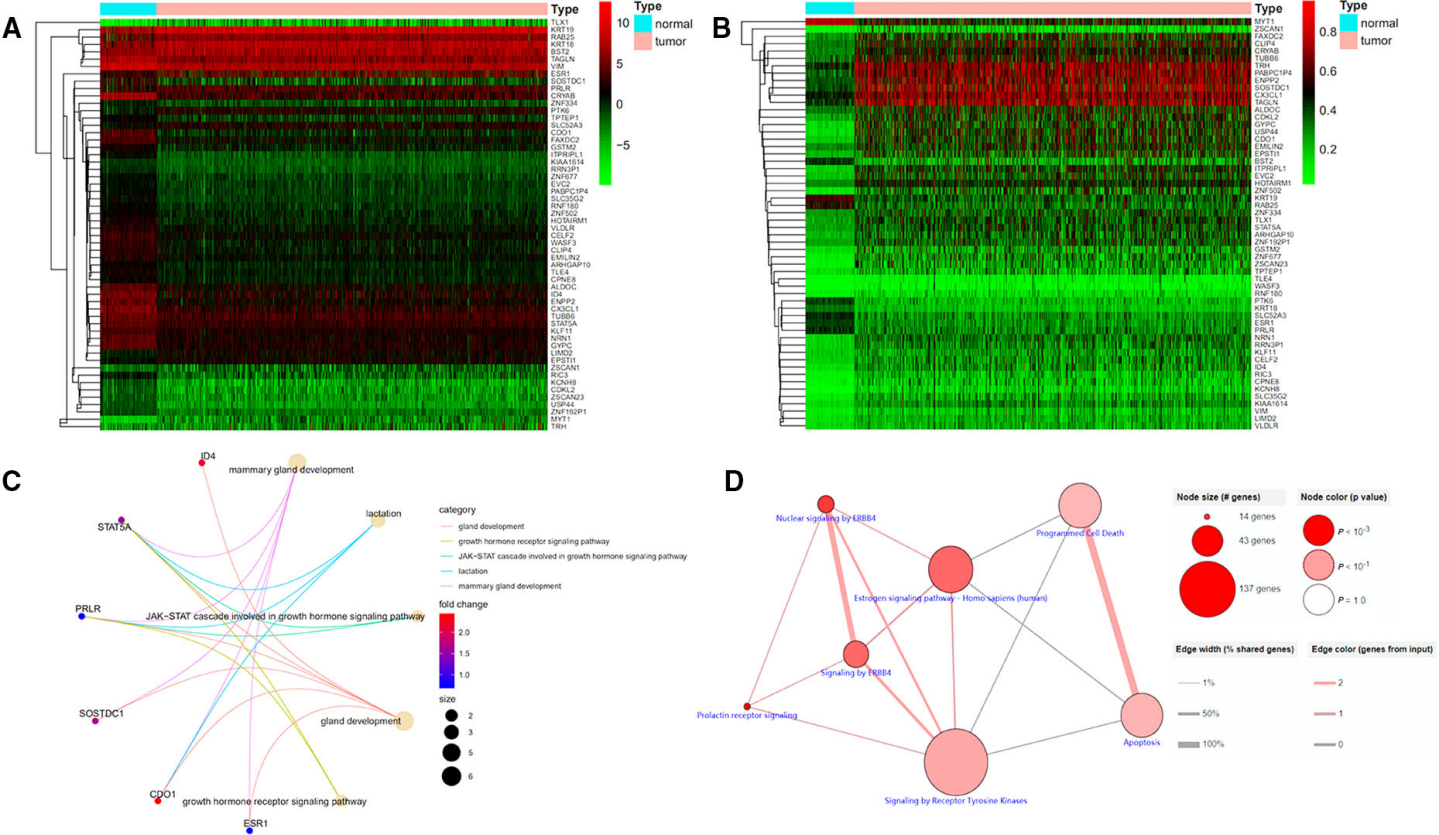
Heatmap of methylation‐driven genes and gene set enrichment analysis. (A) The hierarchical clustering heatmap of RNA expression. (B) The hierarchical clustering heatmap of DNA methylation. (C) GO enrichment analysis. (D) KEGG enrichment analysis.

In total, 56 genes were screened and found to be methylation‐driven genes. Mix models were conducted and performed to determine differential methylation (log_2_ fold change > 0.5, *P* < 0.05, Cor < −0.3). The details of 56 identified genes are shown in Table 1. Mixture models were also plotted and the results indicated that *CDO1*, *CELF2*, *ITPRIPL1*, *KCNH8*, *RIC3*, *USP44*, *ZSCAN1* and *ZSCAN23* were hypermethylated and *PTK6* and *RAB25* were hypomethylated in tumor samples (Fig. 1). The heatmaps of RNA expression (Fig. 2A) and the methylation level (Fig. 2B) of the 56 methylation‐driven genes were plotted with r pheatmap package in r 3.6.1.

**Table 1:** Methylation‐driven genes.

Gene	NormalMean	TumorMean	LogFC	*P*‐value	Cor	Cor *P*‐value
*KRT18*	0.329288565	0.202682784	−0.700128865	5.95 × 10^−50^	−0.46026222	2.62 × 10^−42^
*KRT19*	0.58976045	0.370753249	−0.669669743	1.42 × 10^−49^	−0.458080769	7.09 × 10^−42^
*MYT1*	0.755584988	0.47380599	−0.673297602	7.19 × 10^−48^	−0.426940281	4.96 × 10^−36^
*USP44*	0.141956577	0.458665887	1.691993921	3.78 × 10^−47^	−0.443894753	3.88 × 10^−39^
*HOTAIRM1*	0.291265654	0.450730121	0.629928278	8.56 × 10^−47^	−0.327095945	5.56 × 10^−21^
*PTK6*	0.395896021	0.219152107	−0.853189015	1.25 × 10^−46^	−0.390133469	7.23 × 10^−30^
*PABPC1P4*	0.35120002	0.647831716	0.883326175	1.41 × 10^−46^	−0.54140562	7.76 × 10^−61^
*ESR1*	0.40608298	0.280693027	−0.532781338	1.73 × 10^−45^	−0.632629502	9.11 × 10^−89^
*ITPRIPL1*	0.17655506	0.431960116	1.290779939	6.65 × 10^−45^	−0.535318062	2.89 × 10^−59^
*KIAA1614*	0.148504365	0.296180674	0.995972166	9.98 × 10^−45^	−0.377010511	7.51 × 10^−28^
*TRH*	0.441624806	0.666929094	0.594712177	4.55 × 10^−43^	−0.408073715	8.93 × 10^−33^
*NRN1*	0.165863279	0.339178992	1.032052294	2.69 × 10^−42^	−0.317458357	8.58 × 10^−20^
*ZNF677*	0.098565169	0.310841881	1.657031076	8.88 × 10^−42^	−0.493584344	2.55 × 10^−49^
*GYPC*	0.152755786	0.437924855	1.519456306	1.06 × 10^−41^	−0.350691051	4.48 × 10^−24^
*CRYAB*	0.298461524	0.493277725	0.724855182	7.58 × 10^−41^	−0.498911217	1.63 × 10^−50^
*SLC52A3*	0.436090955	0.282171792	−0.628055295	1.29 × 10^−40^	−0.421303499	4.89 × 10^−35^
*CX3CL1*	0.463518271	0.661910544	0.514010045	9.28 × 10^−40^	−0.502901562	2.02 × 10^−51^
*CDO1*	0.182996461	0.446275462	1.286118734	1.70 × 10^−39^	−0.355442777	9.89 × 10^−25^
*ZNF502*	0.110905161	0.362792848	1.70981951	2.48 × 10^−38^	−0.712226828	3.69 × 10^−12^2
*EVC2*	0.198045554	0.40545554	1.033711409	5.31 × 10^−38^	−0.377961792	5.40 × 10^−28^
*ENPP2*	0.375022803	0.631985993	0.752914261	1.42 × 10^−37^	−0.382410722	1.14 × 10^−28^
*TAGLN*	0.417883713	0.665980116	0.672377575	5.24 × 10^−37^	−0.437915997	5.06 × 10^−38^
*KCNH8*	0.039242282	0.177022811	2.173454448	1.61 × 10^−35^	−0.347504367	1.22 × 10^−23^
*RIC3*	0.087551313	0.22268933	1.346831712	1.79 × 10^−33^	−0.381288788	1.69 × 10^−28^
*RAB25*	0.519788747	0.338474059	−0.618880132	4.94 × 10^−33^	−0.425667179	8.34 × 10^−36^
*ZSCAN23*	0.080664858	0.27486574	1.768714896	2.69 × 10^−32^	−0.348353921	9.32 × 10^−24^
*LIMD2*	0.134231155	0.236071881	0.814506652	3.20 × 10^−32^	−0.367224203	2.09 × 10^−26^
*SOSTDC1*	0.388175221	0.652600903	0.749492958	1.18 × 10^−31^	−0.385249904	4.17 × 10^−29^
*ID4*	0.135846461	0.292724347	1.107565762	6.36 × 10^−30^	−0.381972433	1.33 × 10^−28^
*SLC35G2*	0.100068399	0.251833352	1.331482917	2.01 × 10^−29^	−0.346318429	1.76 × 10^−23^
*TUBB6*	0.329947095	0.532932501	0.691718105	2.40 × 10^−29^	−0.317971477	7.44 × 10^−20^
*PRLR*	0.429700621	0.299950452	−0.518607658	3.27 × 10^−29^	−0.448572298	5.01 × 10^−40^
*CLIP4*	0.313569663	0.496382902	0.662667435	5.01 × 10^−29^	−0.443070142	5.54 × 10^−39^
*TLX1*	0.250818649	0.403427496	0.685664795	1.50 × 10^−28^	−0.366244978	2.90 × 10^−26^
*CDKL2*	0.238525727	0.448014953	0.909402005	2.07 × 10^−27^	−0.433322832	3.52 × 10^−37^
*STAT5A*	0.255622595	0.391453537	0.614825713	1.14 × 10^−25^	−0.639086878	4.14 × 10^−91^
*TLE4*	0.049420326	0.10058383	1.02522197	2.05 × 10^−25^	−0.360809714	1.74 × 10^−25^
*EPSTI1*	0.218238641	0.40452927	0.890337528	1.56 × 10^−23^	−0.335922881	4.16 × 10^−22^
*VIM*	0.10964127	0.207963396	0.923538672	9.05 × 10^−23^	−0.409415054	5.32 × 10^−33^
*BST2*	0.423623437	0.276033797	−0.617937492	4.81 × 10^−22^	−0.449018168	4.12 × 10^−40^
*CPNE8*	0.076972633	0.211402103	1.457572213	5.07 × 10^−22^	−0.388142452	1.48 × 10^−29^
*EMILIN2*	0.290577049	0.449244164	0.628579004	3.19 × 10^−21^	−0.328991988	3.21 × 10^−21^
*RRN3P1*	0.167387934	0.313302674	0.904361542	4.06 × 10^−21^	−0.383765075	7.06 × 10^−29^
*ZNF192P1*	0.248588504	0.374008157	0.589310153	7.68 × 10^−21^	−0.3293247	2.91 × 10^−21^
*FAXDC2*	0.273979021	0.451638118	0.721101827	3.01 × 10^−20^	−0.325571946	8.63 × 10^−21^
*ARHGAP10*	0.212273292	0.359979536	0.761992029	3.73 × 10^−20^	−0.374922274	1.54 × 10^−27^
*VLDLR*	0.149952822	0.235786142	0.652970253	2.93 × 10^−18^	−0.416664173	3.12 × 10^−34^
*KLF11*	0.180346594	0.269535726	0.579704334	1.12 × 10^−17^	−0.322517065	2.07 × 10^−20^
*ZNF334*	0.267950685	0.383554961	0.517465818	5.35 × 10^−17^	−0.339098105	1.60 × 10^−22^
*WASF3*	0.038890285	0.085068153	1.129209331	1.54 × 10^−16^	−0.322775349	1.92 × 10^−20^
*CELF2*	0.176608962	0.294642653	0.738407744	3.91 × 10^−16^	−0.323044228	1.78 × 10^−20^

### Functional enrichment analysis

GO enrichment analysis results (Fig. 2C) showed that gland development, growth hormone receptor signaling pathway, lactation, mammary gland development and the Janus kinase‐signal transducer and activator of transcription cascade in the growth hormone signaling pathway were the most enriched functions. KEGG pathway enrichment analysis (Fig. 2D) showed that prolactin receptor signaling, nuclear signaling by ERBB4, signaling by ERBB4 and the estrogen signaling pathway were the most enriched pathways.

### Combined gene expression and methylation survival analysis

**Fig. 3:**
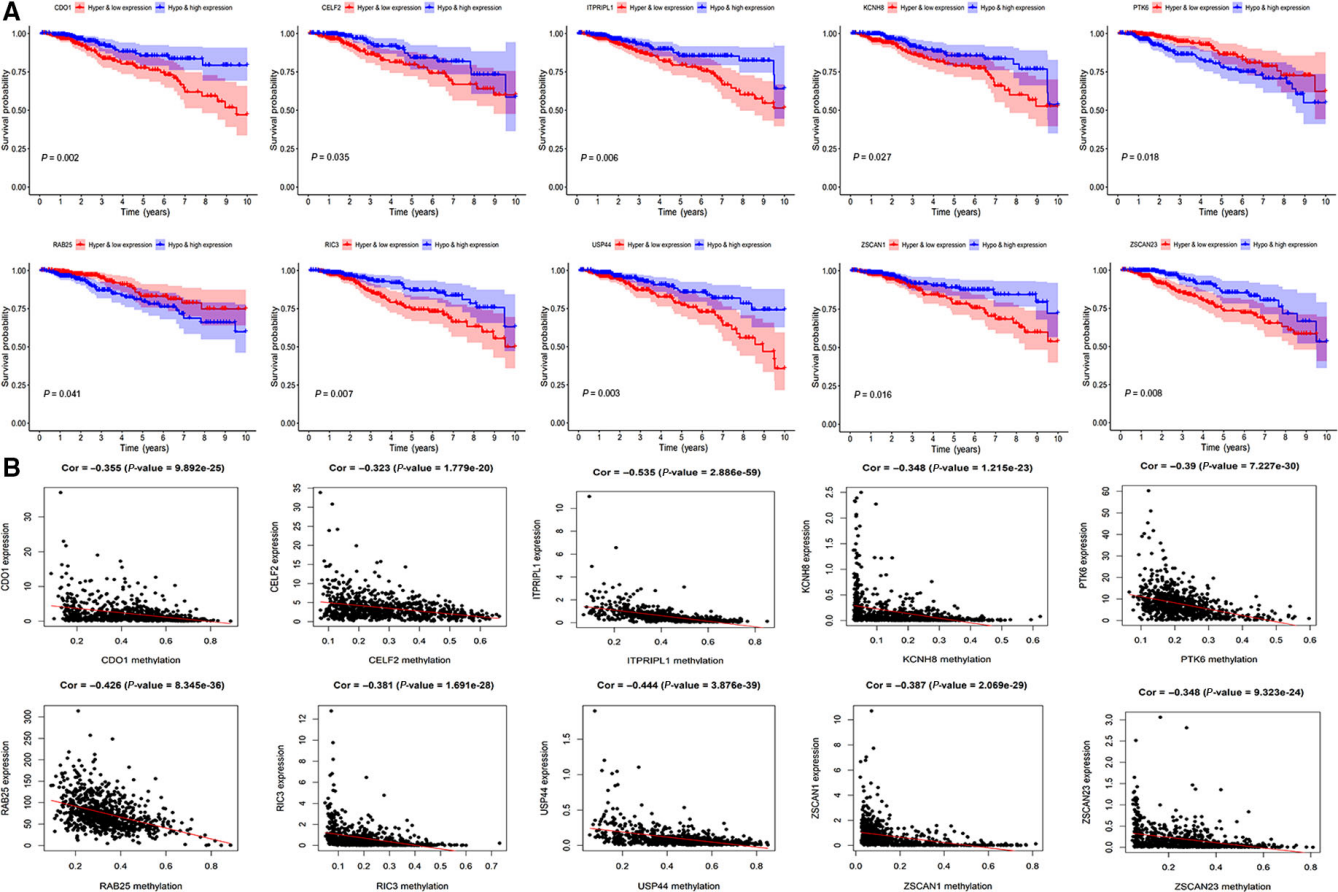
The combined gene expression and methylation data analysis in BRCA. (A) Kaplan–Meier curve analysis of 10 methylation‐driven genes. (B) Pearson correlation analysis between methylation and gene expression. *P* < 0.05 indicates statistical significance.

Kaplan–Meier curves indicated 10 genes were related with the prognosis of BRCA (*P* < 0.05) (Fig. 3A). According to the results, the hypermethylation and low‐expression survival rates of *CDO1*, *CELF2*, *ITPRIPL1*, *KCNH8*, *RIC3*, *USP44*, *ZSCAN1* and *ZSCAN23* were significantly lower. On the other hand, the hypomethylation and high‐expression survival rates of *PTK6* and *RAB25* were significantly lower. Pearson correlation between the DNA methylation level and the RNA expression level revealed that there were significant negative correlations with Cor < −0.3 and *P* < 0.05 (Fig. 3B).

### Identification and analysis of molecular subtypes

**Fig. 4:**
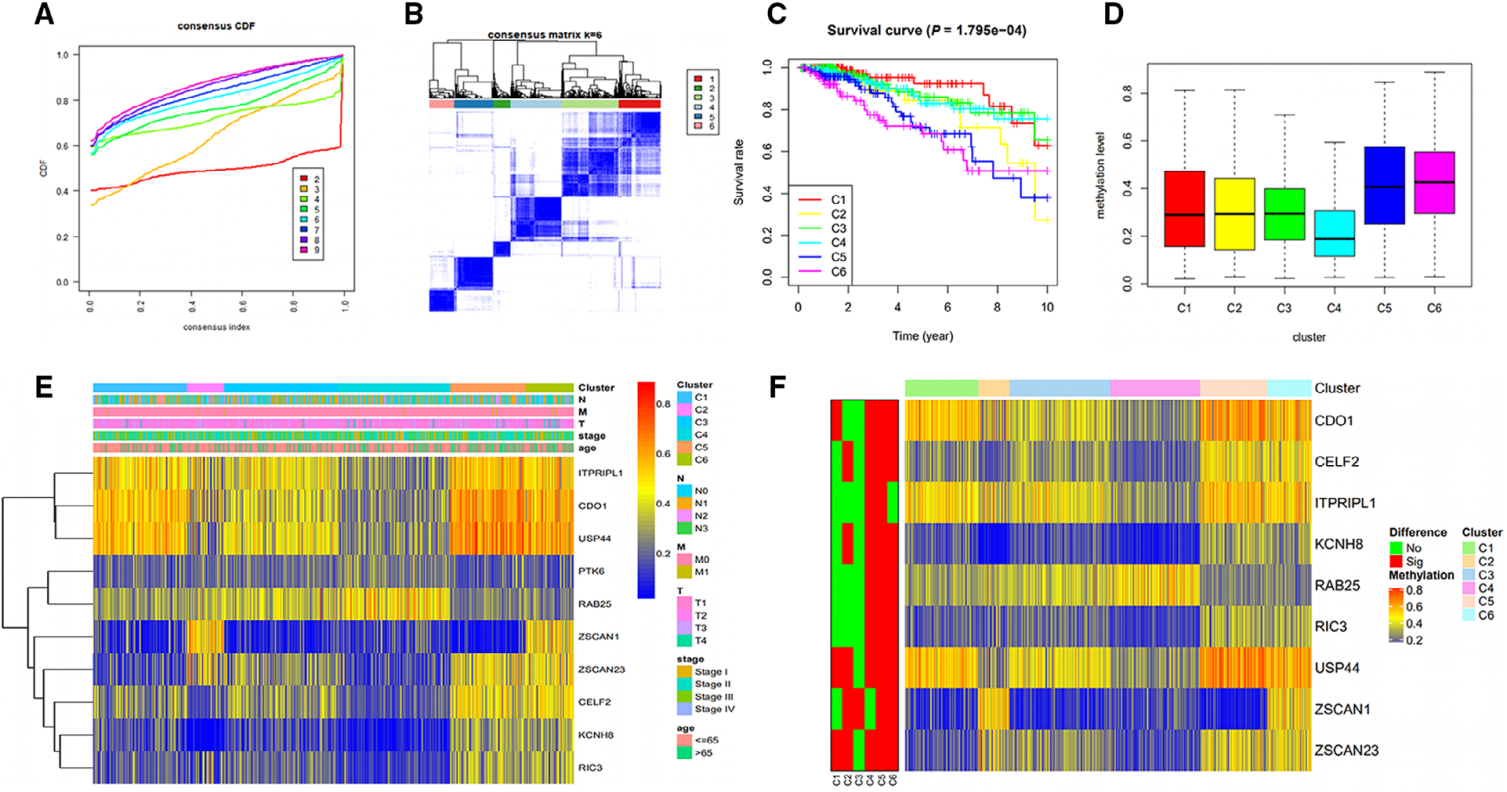
Identification and analysis of molecular subtypes. (A) Consensus among clusters for each category number *k*. (B) Color‐coded heatmap corresponding to the consensus matrix for *k* = 6 obtained by applying consensus clustering. (C) Kaplan–Meier survival analysis for each DNA methylation subtype. (D) Box plot of DNA methylation levels of the six clusters. Cluster 6 has the highest methylation level. (E) A corresponding heatmap of DNA methylation classification, TNM, Stage and Age. (F) Specific hyper/hypo‐methylation‐driven genes for each DNA methylation cluster.

The variations among different clusters and average cluster consensus were estimated to determine the number of clusters. The criteria were followed with higher consistency inside the cluster, lower variations between different clusters and no obvious raise in the area under the cumulative distribution function curve. The area under the cumulative distribution function curve started to stabilize after six clusters (Fig. 4A). The consensus matrix represented the consensus for *k* = 6 and showed a six‐block structure (Fig. 4B). The Kaplan‐Meier plot displayed significant differences among the six clusters (*P* < 0.001) and the clusters 5 and 6 had the worst outcome, whereas cluster 1 had the best survival rate (Fig. 4C).

Different methylation levels of the six clusters were calculated and plotted based on the methylation data of the 10 screened methylation‐driven genes (Fig. 4D). Clusters 6 and 5 showed a higher methylation level than the other clusters, whereas cluster 4 had the lowest level. Associations between clinical features (Age, TNM and Stage) and gene methylation level in different clusters were plotted (Fig. 4E). Different gene methylation levels among clusters were calculated and plotted (Fig. 4F). *USP44* and *ZSCAN23* showed the greatest variant methylation levels, whereas *ITPRIPL1* showed the lowest.

### IHC analysis

**Fig. 5:**
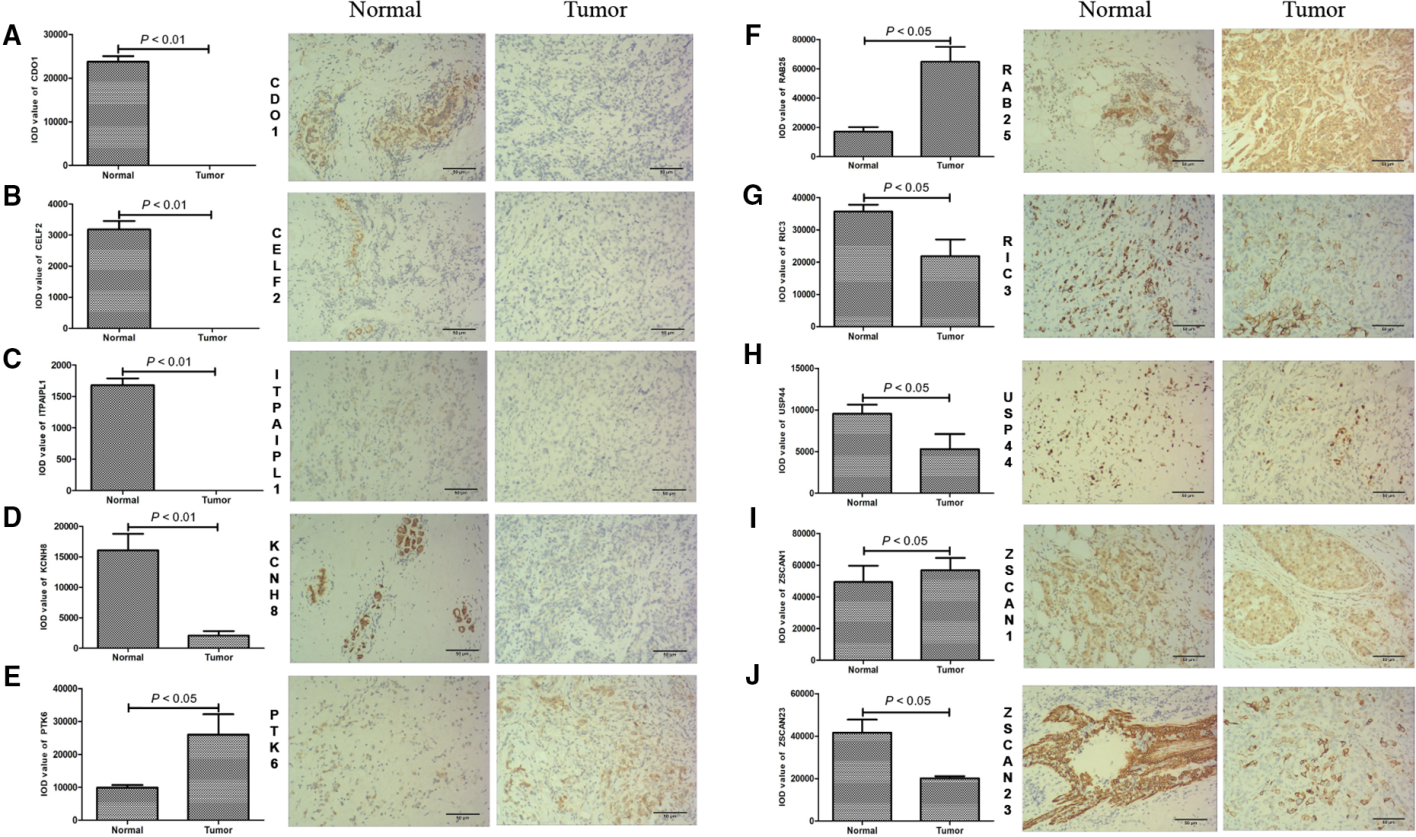
IHC analysis. (A) CDO1. (B) CELF2. (C) ITPRIPL1. (D) KCNH8. (E) PTK6. (F) RAB25. (G) RIC3. (H) USP44. (I) ZSCAN1. (J) ZSCAN23. Integrated optical density (IOD) values in tumor and normal tissues. Paired *t*‐test; *P* < 0.05 indicates statistical significance. Data are the mean ± SD. Representative images at a fixed magnification of 200×. Scale bars = 50 µm.

The protein expression levels of the prognosis methylation‐driven genes were demonstrated by IHC experiments. Representative images of IHC at 200× were obtained (Fig. 5A–J). Clinicopathological characteristics in the present study are shown in Table 2. According to the results, all of the genes except *ZSCAN1* (Fig. 5I) were confirmed to have significantly different expression (*P* < 0.05). Respectively, the expression of CDO1 (Fig. 5A), CELF2 (Fig. 5B), ITPRIPL1 (Fig. 5C), KCNH8 (Fig. 5D), RIC3 (Fig. 5G), USP44 (Fig. 5H) and ZSCAN23 (Fig. 5J) was significantly lower in tumor tissues, whereas the expression of PTK6 (Fig. 5E) and RAB25 (Fig. 5F) was significantly higher in tumor tissues. In the tumor group, CDO1, CELF2 and ITPRIPL1 were not detected, KCNH8 and USP44 were mainly weakly expressed, PTK6, RIC3 and ZSCAN23 were moderately expressed, and RAB25 and ZSCAN1 were strongly expressed. In the normal group, CELF2, ITPRIPL1 and PTK6 were mainly weakly expressed, CDO1, KCNH8, RAB25 and USP44 were moderately expressed, and RIC3, ZSCAN1 and ZSCAN23 were strongly expressed, respectively.

**Table 2:** Clinicopathological characteristics.

Characteristics	Number of cases	% (percentage)
Age		
≥ 60	17	40.48
< 60	25	59.52
Pathological types		
Non‐invasive	3	7.14
Invasive with special type	8	19.05
Invasive with no special type	31	73.81
Tumor stage		
III–IV	29	69.05
I–II	13	30.95
Grade		
III–IV	32	76.19
I–II	10	23.81
Subtype		
Luminal A	19	45.24
Luminal B	10	23.81
Her2^+^	8	19.05
TNBC	5	11.90

### Integration and evaluation of the prognostic nomogram

**Fig. 6:**
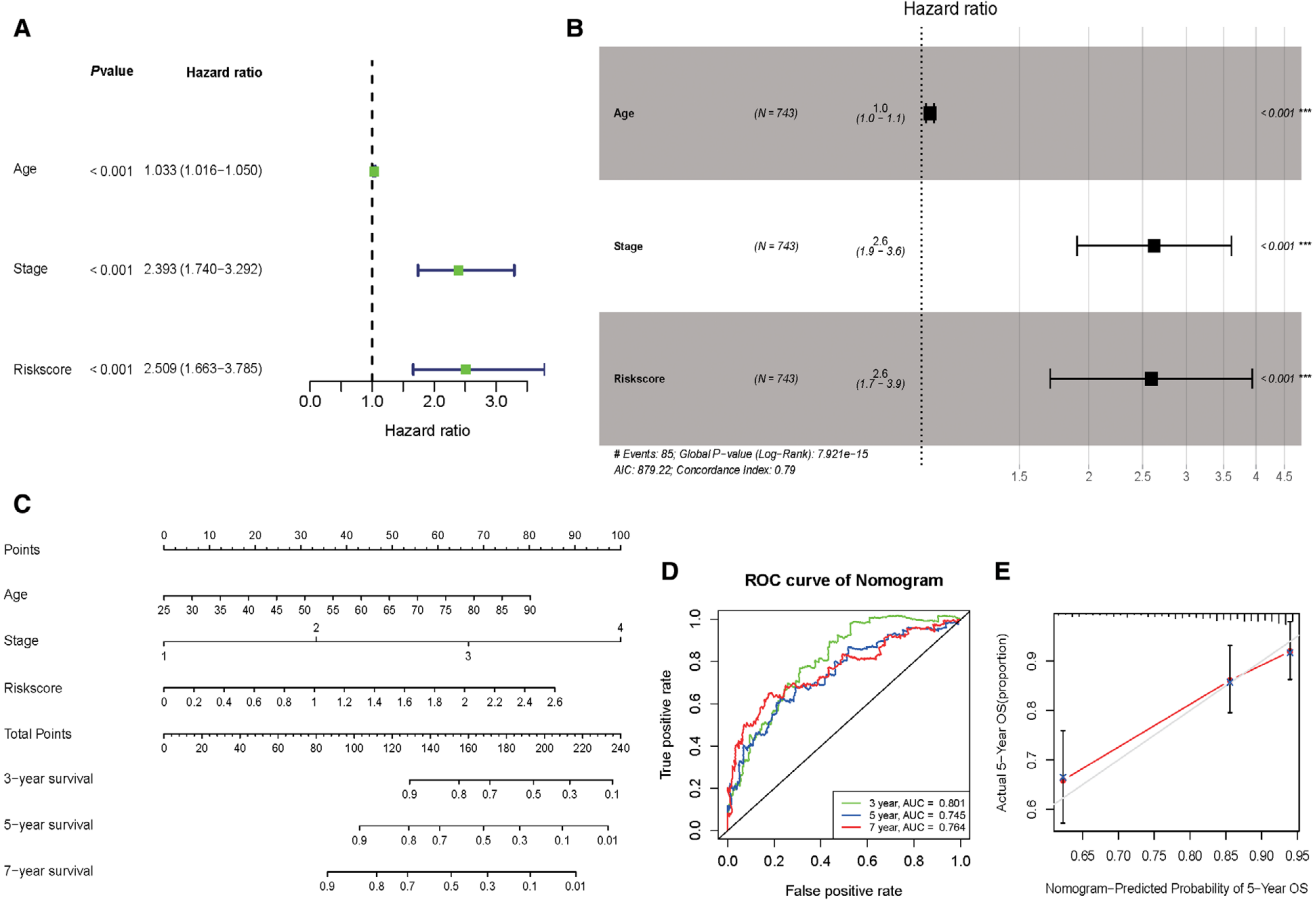
Integration and evaluation of the prognostic nomogram. (A) The *P*‐values and hazard ratios (HRs) in the univariate Cox regression analysis. (B) The *P*‐values and HRs in the multivariate Cox regression analysis. (C) The prognostic nomogram to predict the 3‐, 5‐ and 7‐year overall survival. (D) The ROC curves of 3‐, 5‐ and 7‐year overall survival. (E) The calibration curve of 5‐year overall survival. *P* < 0.05 indicates statistical significance.

After the stepwise regression analysis, three genes were included in the signature calculating the risk score of each patient with the formula: risk score = (−1.01001 ×expression level of ITPRIPL1) + (−0.28248 × expression level of ZSCAN1) + (−0.96202 × expression level of ZSCAN23). All of the three variables (Risk score, Age and Stage) were indicated to be the significant factors (*P* < 0.001) in the univariate and multivariate Cox regression analyses (Fig. 6A,B). A prognostic nomogram incorporating all three significant factors was integrated to predict the 3‐, 5‐ and 7‐year overall survival (Fig. 6C). The 3‐, 5‐ and 7‐year ROC curves were plotted, with an area under the curve of 0.801, 0.745 and 0.764 (Fig. 6D). The calibration curve of 5 years was plotted using the calibrate function and was well calibrated (Fig. 6E).

## Discussion

Recently, DNA methylation has been widely recognized as one important epigenetic modification and is closely related to tumorigenesis. With the development of genomic detection technology, the understanding of the alterations in DNA methylation has greatly improved [13, 14]. Tumorigenesis occurs with extensive DNA methylation changes [15]. Most of these changes happen early in carcinogenesis, which makes DNA methylation valuable for early screening, diagnosis, prognosis, therapeutic classification and follow‐up after treatment [16]. Usually, DNA hypermethylation of tumor suppressor genes or DNA hypomethylation of oncogenes could lead to unfavorable outcomes in patients. In the present study, 56 methylation‐driven genes in BC were screened and 10 of these were associated with the prognosis and tumor classification.

CDO1 (cysteine dioxygenase 1), initiating several key metabolic pathways associated with pyruvate and sulfurate compounds, is an important regulator of cellular cysteine concentrations. In the present study, *CDO1* was a hypermethylated‐low expression gene in BC and this could result in the favorable survival of BC patients. It has been implicated as a novel tumor suppressor gene that is silenced by promoter methylation in many cancers. By analyzing differential RNA expression profiles with or without treatment with 5‐aza‐2′‐deoxycytidine, the frequency of *CDO1* promoter methylation was observed with a statistically significant difference between normal and tumor tissues [17]. In particular, Tanaka *et al*. [18] reported that *CDO1* was silenced at the mRNA level in six types of BC cell lines. Overexpression of CDO1 decreased the growth capacity.

*CELF2* (CUGBP Elav‐like family member 2) is one type of RNA‐binding protein [19]. In the present study, *CELF2* was a hypermethylated‐low expression gene in BC and this could result in the favorable survival of BC patients. Piqué *et al*. [20] demonstrated that *CELF2* was targeted by promoter hypermethylation‐associated transcriptional silencing in BC. The restoration of *CELF2* could inhibit tumor growth and the epigenetic loss induced an aberrant downstream pattern of alternative splicing. Ramalingam *et al*. [21] found that the expression of CELF2 was consistently reduced with neoplastic transformation, indicating that it might be a potential tumor suppressor protein. Yeung *et al*. [22] revealed that CELF2 could interact with PREX2 and reduce the association of PREX2 with PTEN, playing a tumor suppressor role in PI3K signaling by antagonizing the oncogenic effect of PREX2.

*ITPRIPL1* (inositol 1,4,5‐trisphosphate receptor‐interacting protein‐like 1) was a hypermethylated‐low expression gene in BC and this could result in the favorable survival of BC patients. It was identified as a methylation‐driven gene and acted as an independent biomarker for the prognosis of lung adenocarcinoma by using bioinformatics methods [23]. However, little is known of the function and mechanism of *ITPRIPL1* in cancer research.

*KCNH8* (potassium voltage‐gated channel subfamily H member 8) was a hypermethylated‐low expression gene in BC and this could result in the favorable survival of BC patients. It could exhibit RNA polymerase II cis‐regulatory region sequence‐specific DNA binding activity and voltage‐gated potassium channel activity. Using quantitative MethyLight assays, *KCNH8* was found to affect hypermethylation frequencies in lung tumor samples from 117 clinically well‐characterized NSCLC patients [24]. In prostate cancer, *KCNH8* was identified as a novel outlier gene with potential rearrangement and confirmed the association with primary and metastatic prostate samples [25]. However, there are very few studies of *KCNH8* in BC.

*PTK6* (protein tyrosine kinase 6) plays a role as an intracellular signal transducer in epithelial tissues. It was a hypomethylated‐high expression gene in BC and this could result in the unfavorable survival of BC patients. In mammary epithelial cells, overexpression of PTK6 could cause sensitization of the cells to epidermal growth factor and lead to a partially transformed phenotype. Ito *et al*. [26] reported that PTK6 was expressed in approximately 70% of TNBCs and kinase‐active PTK6 suppressed E‐cadherin expression, promoted cell migration and played an important role in promoting an epithelial‐mesenchymal transition. In ER^+^ Luminal BC cells, enhanced expression of PTK6 promoted the growth of ER^+^ BC cells, including tamoxifen‐treated cells [27]. However, another study suggested that the BC cell growth was independent of PTK6 kinase activity. The tumor cell growth inhibition showed no correlation with PTK6 kinase activity inhibition, nor with total or activated PTK6 protein levels [28].

*RAB25* is a member of the RAS oncogene family. In the present study, *RAB25* was a hypomethylated‐high expression gene in BC and this could result in the unfavorable survival of BC patients. Overexpression of RAB25 was correlated with poor prognosis and aggressiveness of renal, lung, breast, ovarian and other cancers [29]. Mitra *et al*. [30] reported that RAB25 was amplified and enhanced aggressiveness in luminal B cancers, whereas, in claudin‐low tumors, RAB25 is lost, indicating possible anti‐tumor functions. In a retrospective study, the expression of RAB25 was evaluated by IHC in 57 primary BC samples. The results obtained indicated that the expression of RAB25 was correlated with clinicopathologic variables and different molecular subtypes[31].

*RIC3* (RIC3 acetylcholine receptor chaperone) was a hypermethylated‐low expression gene in BC and this could result in the favorable survival of BC patients. It could encode a member of the resistance to inhibitors of the cholinesterase 3‐like family, which may have functions including inflammation control. In human lymphocytes and macrophages, immune activation could lead to dynamic changes in RIC3 expression and RIC3 was found to show a strong correction with inflammatory processes [32]. In another study, RIC3‐TCRBC2 fusion was identified by RNA‐Seq in T‐cell lymphoblastic lymphoma, which might be a strong driver for neoplasia‐associated mutations [33]. No study has yet focused on the relationships of RIC3 and any other cancer.

*USP44* (ubiquitin carboxyl‐terminal hydrolase 44) is a protease that functions as a deubiquitinating enzyme. The present study indicated that it was a hypermethylated‐low expression gene in BC and this could result in the favorable survival of BC patients. *USP44* was identified as a key regulator of anaphase‐promoting complex activation [34]. In BC, Liu *et al*. [35] reported that *USP44* silencing induced spindle multipolarity, abated vasculogenic mimicry, reduced transendothelial migration and decreased interleukin‐6 and interleukin‐8 levels in BC stem cells. Lan *et al*. [36] indicated that *USP44* contributed to N‐CoR functions with respect to regulating gene expression and was required for the efficient invasiveness of TNBC cells. Sloane *et al*. [37] reported that the *USP44* CpG Island was hypermethylated in colorectal cancer cell lines.

*ZSCAN1* (zinc finger and SCAN domain‐containing protein 1) and *ZSCAN23* (zinc finger and SCAN domain‐containing protein 23) belong to the same gene family. In the present study, these two genes were hypermethylated‐low expression genes in BC and this could result in the favorable survival of BC patients. However, the protein level of ZSCAN1 was not found to demonstrate a significant difference. On the whole, the understanding of these two genes is quite limited. In a study identifying DNA methylation markers for the detection of high‐grade cervical intraepithelial neoplasia, the SOX1/ZSCAN1 panel (84%, 167/200) had a higher sensitivity and specificity compared to the others [38]. The role of ZSCAN23 in cancer has not yet been reported.

With these 10 survival‐related and methylation‐driven genes, a signature estimating the risk score of each patient was constructed with stepwise regression based on the RNA‐Seq data. It was confirmed to be an independent prognosis factor by univariate and multivariate Cox regression analyses. A prognostic nomogram including over 700 BC patients was further integrated to predict the 3‐, 5‐ and 7‐year overall survival. The area under the curve values of the 3‐, 5‐ and 7‐year ROC curves were 0.801, 0.745 and 0.764, which demonstrated good prediction ability.

In conclusion, the DNA methylation levels of these 10 methylation‐driven genes (*CDO1*, *CELF2*, *ITPAIPL1*, *KCNH8*, *PTK6*, *RAB25*, *RIC3*, *USP44*, *ZSCAN1* and *ZSCAN23*) were negatively correlated with the mRNA expression level after linear regression analysis with the MethylMix r package. Using the ConsensusClusterPlus r package, six subgroups were identified with a significantly different prognosis based on the methylation data. The protein levels were confirmed by IHC and nine of 10 (i.e. except ZSCAN1) showed statistical differences. The prognosis values of the 10 identified methylation‐driven genes in BC were explored. Finally, a prognostic nomogram including over 700 BC patients was further integrated to predict the 3‐, 5‐ and 7‐year overall survival with good prediction ability. Taken together, the screened methylation‐driven genes could be potential biomarkers of BC.

However, there are also some limitations to the present study. For example, the results of the study are mainly based on the bioinformatic analysis. The DNA methylation changes, gene functions and mechanisms of the methylation‐driven genes could be better revealed in additional experiments. Moreover, the prediction ability of the integrated prognostic nomogram requires more research to be validated for clinical practice.

## Conflict of interests

The authors declare that they have no conflicts of interest.

## Author contributions

XZ conceived and designed the project. XZ and GZ acquired the data. XZ and GZ analyzed and interpreted the data. XZ wrote the paper.

## Data Availability

All data included in this study are available upon request by contact with the corresponding author.
